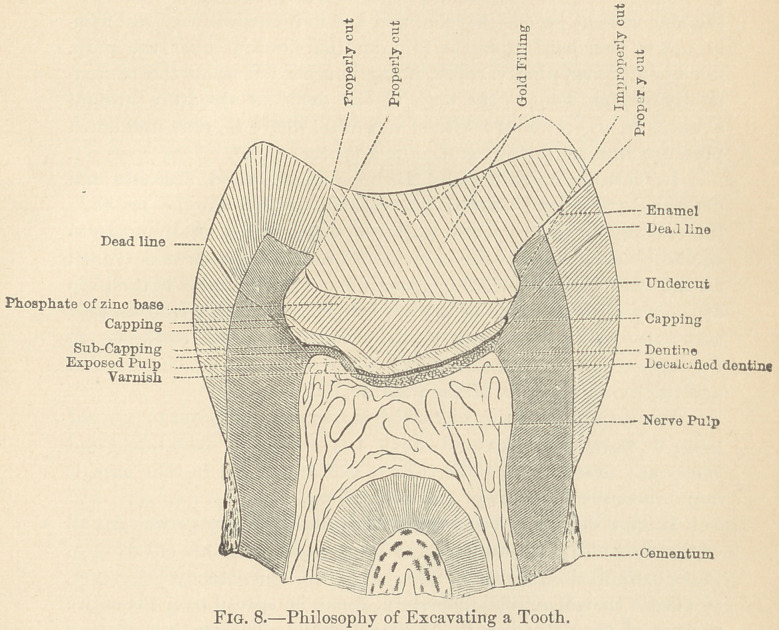# Retaining Plugs Used in Regulating

**Published:** 1889-05

**Authors:** J. N. Farrar

**Affiliations:** New York


### RETAINING PLUGS USED IN REGULATING.*

### Wire Retaining Pegs.—Wart Retaining Fillings.—Philosophy of Excavation.†

BY J. N. FARRAR, M.D., D. D. S., NEW YORK.

For some cases, if nicely made, these auxiliaries may be classed among the very best devices for retaining purposes. As their practicability depends so much on the manner of their construction they will be explained in detail. Beginning with the consideration of pins set in cavities we will then proceed to the consideration of the wart-plugs.

*Wire retaining Pegs.*—Pins to be fixed into cavities are of gold or platinum wire, one extremity of which is roughened or screw-cut and the other smooth. When there are natural cavities, pins are easily set with cement; but if the holes are to be drilled for the purpose it becomes a matter of nice calculation, for they should not be so large as to make it necessary to have a ring of cement around the pin to be exposed, nor should they be so small that the threads of the screw will crumble the enamel; but they should be sufficiently large to permit the pin to enter snugly. Figs. 1 and 2 illustrate sectional views of lower cuspid teeth, showing the

---

* Advance sheets from Dr. Farrar's work on "Irregularities of the Teeth," published in the INTERNATIONAL by special permission of the author.

† Copyrighted.

relation of the pin, J, to the enamel, E, dentine, F, and pulp, P. The figure at the top shows the philosophy of the leverage; D, fulcrum, H, resistance or weight, arrow; the power or point upon which the regulated tooth bears.

It is very important that the bearings of the screw or pin, when pressed against by the recently moved tooth, B, as indicated by the arrows, should rest against the tooth tissue at the entrance at D, Fig. 2, and also at the bottom of the cavity at H, and although

FIGS. 1 and 2.—Screw Retaining-pegs (A).

in contact, not to rest on the cement in which it is set. It is necessary in order to obtain firmness, however, to shape the pit so that there will be a little extra room in other places for a small quantity of cement, as indicated in black. When the dentine is thick enough, this chamber should be made entirely within it, as shown in Fig. 2, not sacrificing the enamel, as shown in Fig. 1. In this way the fulcrum, which is the enamel, D, will be strong and not crumble. After the corrected teeth have been retained in place long enough the pegs should be removed and the cavities filled with gold.

*Wart retaining Plugs.*—When teeth overlap, they may sometimes be retained in position after correction by simply inserting wart-plugs in natural cavities, if there are any in the approximal surfaces or in artificial cavities if the urgency of the case demands it, which is seldom. Fig. 3 illustrates the principle of wart-plugs, which, if rounded off and polished, are not objectionable. Indeed, I never saw, except under circumstances hereafter to be explained, more satisfactory results from any kind of retainer, because these

Fig. 3.—Wart Filling for Retaining Purposes (A).

may remain permanent without disfigurement or inconvenience. The durability of wart-plugs depends entirely upon the skill exercised in the operation; "no slip-shod" method of inserting gold will be effectual. The plug should be made of adhesive gold inserted after the laminated method of filling teeth—that is, the foil should lie like the leaves of a book, one piece flat upon another, beginning at the posterior part of the cavity and extending outward.

Fig. 4.—Longitudinal Section View of a Tooth and Wart-plug (A).

Fig. 4 illustrates a longitudinal section view of a central incisor having a natural cavity, showing this laminated construction of a gold wart-plug, as made by the author.

Fig. 5, a transverse sectional view of the same tooth cut in line of x, x, illustrates the same—L, the plug; E, enamel; D, dentine; P, pulp; S, D, calcified portion of pulp, ("secondary dentine"); C, capping material. The wavy lines shown in the plug, L, in Fig 4, indicate the relation of the layers of gold with the tooth.

Fig. 5.—Transverse Section View of a Tooth with Wart-plug.

Beginning the operation in the usual way against the cervical wall of the cavity with soft foil, shown in black in Fig. 4, it is followed by piece after piece of laminated gold, cut either from a carefully prepared roll, or better, from ribbon made of several thicknesses of cohesive sheets (perhaps heated). These are malleted together at all points, but more especially at the outer ends of the pieces, in order to bend them toward the gum, and give a solid surface. When the cavity is nearly filled, a key, K, of roll or rope foil, is forced into the undercut, as shown by curved lines. This key renders the wart-plug very secure, and when all is properly finished and polished, it is not objectionable to the eye.

Fig. 6.—Central Incisor prepared for Filling (front view).

Fig. 6 illustrates the appearance of the anterior surface of a central incisor as prepared for the filling. This method of cutting a hatchway through the anterior portion of the tooth renders the operation of filling easier than to operate from underneath, and also leaves little or no disfigurement of the tooth. It is commonly thought that a filling should never show if possible; but this opinion is not always sound, for gold thus inserted between the teeth often reflects a dark line to the eye which, had the gold been made to extend a short distance around on the anterior surface of the tooth, would form in the light a bright line which would catch the eye and render the dark line beyond unobserved. This is a form of plug that is not only more durable, but causes no disfigurement, and is not very noticeable.

FIG. 7.—Wart-plug (section view of tooth only).

Fig. 7 illustrates a longitudinal section of a central incisor having two wart-plugs. The tooth is represented as divided after the plugs were inserted, so as to show the relation of the outer surface of the wart-plug with the inner portion and the inner parts of the tooth. In other words, the anterior half of the tooth is removed, leaving the plugs intact. The external surface of the plug, L′, L′, is shown in black, the internal, J, J, in lines. This figure also illustrates the form of the cavities.

*Philosophy of Excavation minutely considered.*—In the preparation of the cavity it probably is not necessary to mention to the majority of operators that to excavate in accordance with the "cleavage," or "grain," so to speak, of the enamel, is an important point, and one which should not be lost sight of. But as it may be instructive to students, an extract from a paper by the author, published in January, 1879,[1] is given here. As this point cannot be so clearly shown in a diagram of an incisor, it will be illustrated in a section view of a molar, drawn not only to show the philosophy of proper excavation of a cavity, but the details of management of the worst conditions, including an exposed pulp (Fig. 8).

FIG. 8.—Philosophy of Excavating a Tooth.

In this figure the position of the enamel rods is illustrated by lines radiating from the dentine, and the heavier lines on the margin of the cavity show how the enamel may be cut without weakening and rendering it liable to "chip out" around the filling. It also shows by other lines where the rods may, and also where they should not be, diagonally cut, in order to prevent chipping. This would occur if they were cut along the dotted line on the right, but would not if cut as shown on the left.

In preparing the dentinal portion of the cavity when the pulp is exposed, it is well also to cut away the walls and floor, so that not

[1] "Missouri Dental Journal," January, 1879.

only the filling, as a whole, may be firmly anchored, but that the ends of all dentinal tubes, leading directly from the cavity to the pulp, may be covered with varnish (the best kind of which is fir balsam dissolved in chloroform).[1] But to accomplish this double object, all cavities in the grinding surfaces of teeth, where great pressure is to be made, should, if possible, be so shaped that when the zinc cement base is inserted and has hardened, it may rest firmly, not to bear hard upon the thin dentinal flooring over the pulp, nor upon the capping varnish, if any is used, but upon the centrifugally inclined floor of the walls of the dentinal structure around about. In approximal cavities, however, where there is not much pressure applied, this precaution is not necessary.

In case of exposed pulp, having applied wood creosote and deposited the proper quantity of varnish—which should be very limited—upon the exposed point, it should be carefully made to spread over the floor of the cavity with a delicate instrument until all the dentinal tubes leading from the cavity directly to the pulp are covered, in order, as before implied, to cut off all irritation that may cause pain. This may be easily understood by reference to the figure, where the dead lines show the division between the dead and the living portions of the dentine and enamel.

While the application of varnish is important in order to prevent pain, it should not extend much beyond the "dead-line," and smear any more of the walls than is necessary; for in that case it may injure the firmness of the main filling.

Having deposited the varnish, it is not necessary to wait for all of the chloroform to evaporate before applying the thin covering of phosphate of zinc, which should be of the consistency of cream, so plastic that, if applied delicately, it can be spread over the entire surface of the varnish without displacing it. The smooth coating of varnish is soothing to the pulp and insulates it, as it were, from the cement. Should, however, the varnish coating be injured, pain will generally follow in proportion to the extent of the injury of the insulator. This varnish prevents disintegration of the cement, which is sure to follow in time, if the juices of the pulp are directly exposed to it.

Having the pulp protected, and the varnish hermetically sealed in by the cement, the chloroform is retained and made useful by

---

[1] The genuine fir balsam is not often found in market; the imitation is pine pitch, which will not harden, therefore is not fit for capping purposes. I get fir balsam direct from the forest by gathering the "tears" that ooze from cuts in the trees.

acting upon the exposed portion of the pulp beneath; and the morphine mixed with the wood creosote acting upon that portion of the crown of the pulp which is not exposed, the entire pulp is soon rendered sufficiently insensible to any slight irritation that may take place—a condition tending to obviate any "shock" and which will continue until nature has time to act and becomes habituated to the new environments—a point of great value to both patient and operator.

Having accomplished the important point of painlessly protecting the pulp, a proper additional amount of cement, harder than that used for the flooring, should be introduced, after which, when it is sufficiently hardened, it may be dressed and shaped in readiness to serve as a basis or foundation upon which to build a wart plug. This operation, though applicable to approximal cavities in the front teeth when it is possible to carry it out, is especially valuable to molars requiring wart-plugs for keeping the jaws apart while "jumping" a tooth, or in operations for correcting protruding teeth where the lower incisors are so long that they bear upon the upper teeth.

## Figures and Tables

**Figs. 1 and 2. f1:**
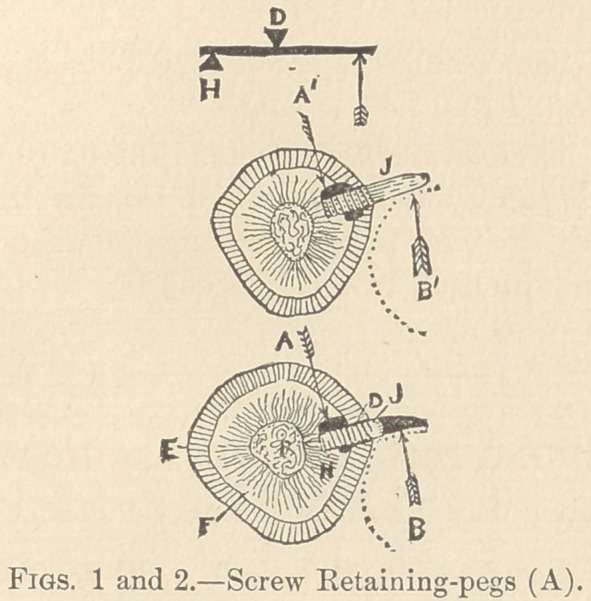


**Fig. 3. f2:**
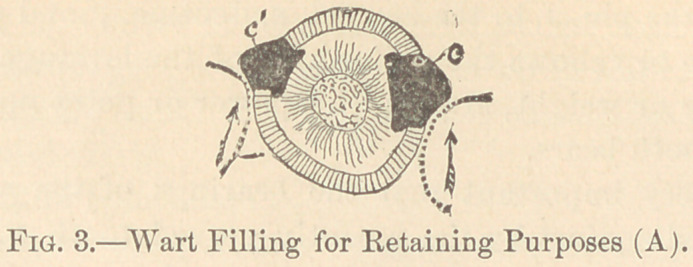


**Fig. 4. f3:**
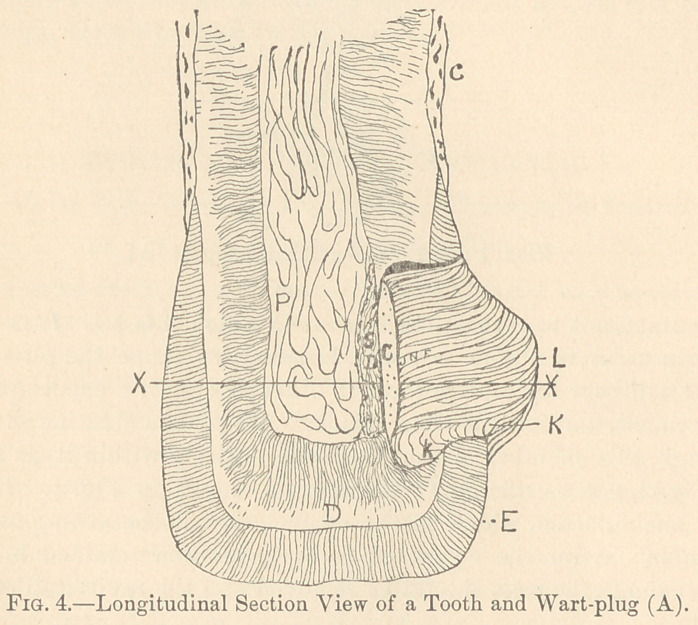


**Fig. 5. f4:**
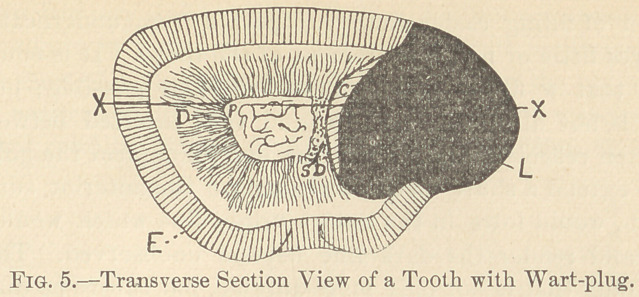


**Fig. 6. f5:**
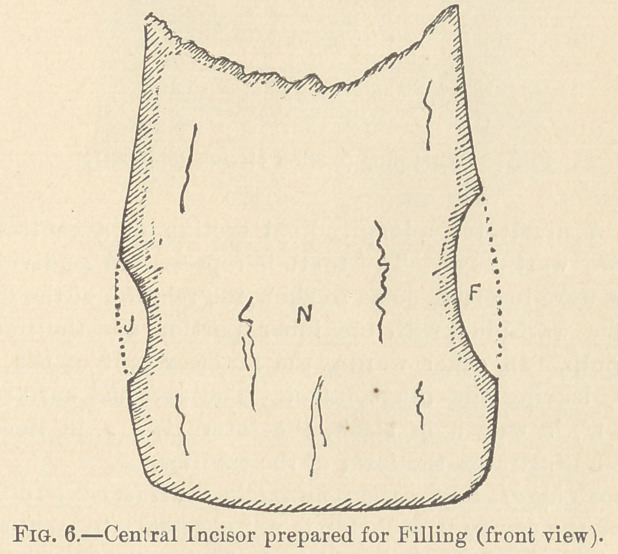


**Fig. 7. f6:**
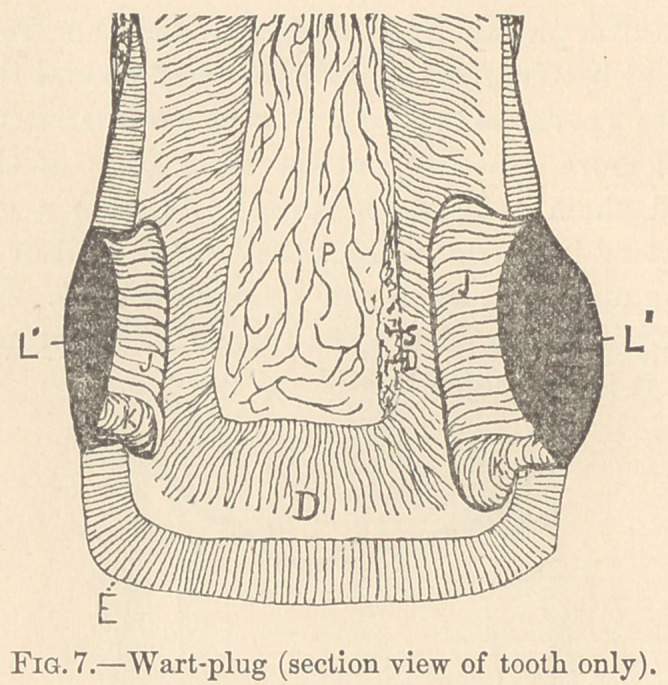


**Fig. 8. f7:**